# Association of *MIAT* genetic variants and expression with tumor grade in oral tongue cancer

**DOI:** 10.7150/ijms.129445

**Published:** 2026-02-26

**Authors:** Ping-Ju Chen, Chiao-Wen Lin, Chun-Yi Chuang, Shun-Fa Yang, Ying-Erh Chou

**Affiliations:** 1 Department of Dentistry, Changhua Christian Hospital, Changhua, Taiwan.; 2 Department of Post-Baccalaureate Medicine, College of Medicine, National Chung Hsing University, Taichung, Taiwan.; 3 Institute of Medicine, Chung Shan Medical University, Taichung, Taiwan.; 4 Institute of Oral Sciences, Chung Shan Medical University, Taichung, Taiwan.; 5 Department of Dentistry, Chung Shan Medical University Hospital, Taichung, Taiwan.; 6 School of Medicine, Chung Shan Medical University, Taichung, Taiwan.; 7 Department of Otolaryngology, Chung Shan Medical University Hospital, Taichung, Taiwan.; 8 Department of Medical Research, Chung Shan Medical University Hospital, Taichung, Taiwan.

**Keywords:** oral cancer, MIAT, polymorphism, cell differentiation, betel-quid chewing

## Abstract

Oral tongue cancer is an aggressive malignancy and represents the most common subsite of head and neck cancer. The long non-coding RNA myocardial infarction associated transcript (MIAT) has been implicated in the development and progression of several cancers. This study aimed to investigate the association between *MIAT* single-nucleotide polymorphisms (SNPs) and oral cancer susceptibility as well as related clinicopathological characteristics. In this study, *MIAT* SNPs (rs4274, rs1061540, and rs1894720) were genotyped using real-time polymerase chain reaction in 1,194 controls and 397 male patients with tongue cancer. A significant association was observed between the *MIAT* rs4274 AA genotype and the occurrence of tongue cancer compared with controls (p = 0.018). Among the patients, carriers of the rs4274 A allele exhibited a higher likelihood of developing moderately or poorly differentiated tumors [OR (95% CI) = 2.246 (1.217-4.147); p = 0.001]. Betel-quid chewers and smokers carrying the A allele showed similarly elevated risks for poor tumor differentiation. Bioinformatic analyses further indicated that the rs4274 A allele is associated with increased MIAT expression, and higher MIAT levels correlated with higher tumor grade. In conclusion, the *MIAT* rs4274 A allele is linked to poorer tumor differentiation, particularly among betel-quid chewers and smokers, and elevated MIAT expression supports its potential as a biomarker of tumor aggressiveness.

## Introduction

Oral cancer is a major subtype of head and neck malignancies, encompassing cancers of the tongue, lips, oral cavity, and oropharynx 1, 2. Among these, oral tongue squamous cell carcinoma (OTSCC) is the most prevalent, accounting for 25-40% of oral cancer cases 3, 4. Exposure to environmental risk factors, including tobacco smoking, alcohol consumption, and betel quid chewing, has been identified as a significant contributor to oral carcinogenesis 5-8. Despite extensive research, it remains unclear whether the functional consequences of genetic alterations are consistent across OTSCC. Consequently, further studies are warranted to evaluate the reproducibility and clinical significance of these genetic phenotypes in OTSCC.

Myocardial infarction associated transcript (MIAT) was first identified as a novel long non-coding RNA significantly associated with myocardial infarction (MI) on chromosome 22q12.1 9. In OTSCC, upregulated MIAT was shown to promote epithelial-mesenchymal transition (EMT) in OTSCC cells via activation of the Wnt/β-catenin signaling pathway 10. Furthermore, several studies have investigated MIAT expression in oral squamous cell carcinoma (OSCC) and suggested that it may serve as a potential prognostic biomarker, reflecting its possible role in disease progression and patient outcomes 11, 12. In addition, *MIAT* polymorphisms have been reported to be associated with cancer development and progression across multiple malignancies 9, 13-21. For example, in ovarian cancer, the C/C genotype of *MIAT* rs1061540 was significantly more frequent in malignant epithelial tumors compared to low-grade tumors, whereas the T/T genotype was linked to a lower risk of malignancy, suggesting that specific *MIAT* genetic variants may influence tumor behavior and patient prognosis 14. However, the associations between *MIAT* polymorphisms and oral tongue cancer progression, as well as clinicopathologic characteristics, remain unexplored. In this study, we examined three SNPs of *MIAT* rs4274, rs1061540, and rs1894720, and try to elucidate their associations to oral cancer susceptibility and clinicopathologic characteristics with environmental risk factors.

## Materials and Methods

### Study subjects

A total of 1194 cancer-free controls and 397 male patients with tongue cancer were participated in our study. All participants in this study were enrolled at Chung Shan Medical University Hospital, Taichung, Taiwan. Tumor differentiation in male patients with tongue cancer was evaluated and graded by pathologists according to the American Joint Committee on Cancer (AJCC) classification system. In addition, TNM staging of the male tongue cancer patients included in this study was clinically determined at the time of diagnosis in accordance with the AJCC guidelines. This project was approved by the institutional review board of Chung Shan Medical University Hospital (CSMUH No: CS1-21151). The informed written consent was provided to each patient who enrolled in this study. The control group who participated in our study was those individuals who without self-reported diseases such as asthma, autoimmune and neurological diseases, cardiovascular diseases, diabetes, and history of cancer of any sites.

### Sample preparation and DNA extraction

Peripheral blood samples from normal controls and oral tongue cancer patients participating in this study were collected for genomic DNA extraction 22. Each sample was preserved in EDTA-containing tubes. The blood samples were centrifuged at 3,000 rpm for 10 minutes, and the resulting buffy coats were collected for DNA extraction. Genomic DNA was isolated using the QIAamp DNA Blood Mini Kit according to the manufacturer's instructions. The final elution was performed with Tris-EDTA (TE) buffer. The extracted DNA was subsequently used as a template for real-time polymerase chain reaction (PCR) analyses.

### Selection of *MIAT* SNPs

In this study, three *MIAT* SNPs rs4274, rs1061540, and rs1894720 were selected based on data from the International HapMap Project database 23. The *MIAT* rs4274 polymorphism was chosen because carriers of the AA genotype have been reported to have an increased risk of paranoid schizophrenia in a Chinese Han population 21. The *MIAT* rs1061540 polymorphism was selected due to its reported association with the severity of coronary artery disease (CAD) and the Gensini score in an Egyptian population 24. In addition, a study on ovarian cancer demonstrated that malignant epithelial tumors exhibited a higher frequency of the *MIAT* rs1061540 C/C genotype compared with low-grade epithelial tumor cohorts 14. Regarding *MIAT* rs1894720, a study investigating ischemic stroke (IS) susceptibility in a Chinese population reported that individuals carrying the GG genotype had a significantly increased susceptibility to the large-artery atherosclerosis (LAA) subtype compared with healthy controls. Moreover, carriers of the rs1894720 GG genotype exhibited higher serum MIAT levels than those with the GT or TT genotypes 25.

### *MIAT* SNPs genotyping

Assessment of allelic discrimination for the *MIAT* rs4274 (assay IDs: C__11476158_20), rs1061540 (assay IDs: C___2467719_1_), and rs1894720 (assay IDs: C__11476152_10) SNP was performed with an ABI StepOne Software v2.3 Real-Time PCR System. The TaqMan assay was applied for the analysis of genotyping 26. The analysis and calculation of the collected data of genotyping was processed with the SDS 7000 series software (Applied Biosystems, Foster City, CA, USA).

### Published databases for validation

Multiple publicly available databases were employed to validate our findings. The Genotype-Tissue Expression (GTEx) portal provides comprehensive data on human gene expression, regulation, and genetic variation, which we used to assess MIAT expression and the SNP rs4274 27. The University of Alabama at Birmingham CANcer (UALCAN) database offers an interactive platform for analyzing cancer OMICS data from TCGA, allowing us to examine MIAT expression in relation to clinicopathological features of head and neck squamous cell carcinoma (HNSC) 28.

### Statistical analysis

To compare the age, betel quid chewing, cigarette smoking, alcohol drinking, tumor stage, tumor T status, lymph node status, metastasis, and cell differentiation between the oral tongue cancer patients and the controls, the student's t test or Chi-squared test was applied to evaluated the data in these two groups. A p < 0.05 was considered to represent statistical significant. To analyze the odds ratio (OR) with their 95% confidence intervals (CIs) of the association between the genotypic frequencies of *MIAT* and the clinical pathological statuses in oral tongue cancer patients, the multiple logistic regression models was introduced for the analysis of the data. The SAS statistical software (Version 9.1, 2005; SAS Institute, Cary, NC) was used to evaluate all the data analysis in our study.

## Results

The distribution of demographic characteristics among 1,194 controls and 397 male patients with tongue cancer is presented in Table 1. In this study, the proportion of participants aged <55 years was 564 (47.2%) in controls and 192 (48.4%) in male tongue cancer patients, whereas the proportion aged ≥55 years was 630 (52.8%) in controls and 205 (51.6%) in patients. The distributions of environmental risk factor exposures differed significantly between controls and male tongue cancer patients: 198 (16.6%) vs. 264 (66.5%) for betel quid chewing (p < 0.001), 634 (53.1%) vs. 307 (77.3%) for cigarette smoking (p < 0.001), and 237 (19.9%) vs. 149 (37.5%) for alcohol consumption (p < 0.001).

The adjusted odds ratios (AORs) and 95% confidence intervals (CIs) for tongue cancer associated with *MIAT* genotype frequencies are shown in Table 2. Among patients with tongue cancer, the most frequent *MIAT* genetic polymorphisms were rs4274 G variant, rs1061540 T variant, and rs1894720 G variant. After adjusted for betel quid chewing, cigarette smoking, and alcohol consumption. A significant association was observed between tongue cancer and the *MIAT* rs4274 “AA” genotype (p = 0.018, Table 2).

We further analyzed the ORs and 95% CIs of clinical characteristics according to *MIAT* rs4274 genotypes in male tongue cancer patients. A significant association was identified between *MIAT* rs4274 polymorphisms and tumor differentiation grade [OR (95% CI) = 2.246 (1.217-4.147); p = 0.001] (Table 3). Additionally, when stratifying male tongue cancer patients by betel quid chewing status, betel quid chewers carrying the *MIAT* rs4274 “GA+AA” variants had a higher risk of moderate or poor tumor differentiation [(OR) 2.968; 95% CI: 1.374-6.410; p = 0.006] (Table 4). Among smokers, those with the “GA+AA” genotype similarly exhibited an increased risk of moderate or poor differentiation [(OR) 2.867; 95% CI: 1.405-5.849; p = 0.004], whereas non-smokers carrying the same genotype showed a significantly higher risk of developing advanced clinical stage (stage III+IV) [(OR) 2.704; 95% CI: 1.087-6.726; p = 0.033] (Table 5).

**Figure 1:**
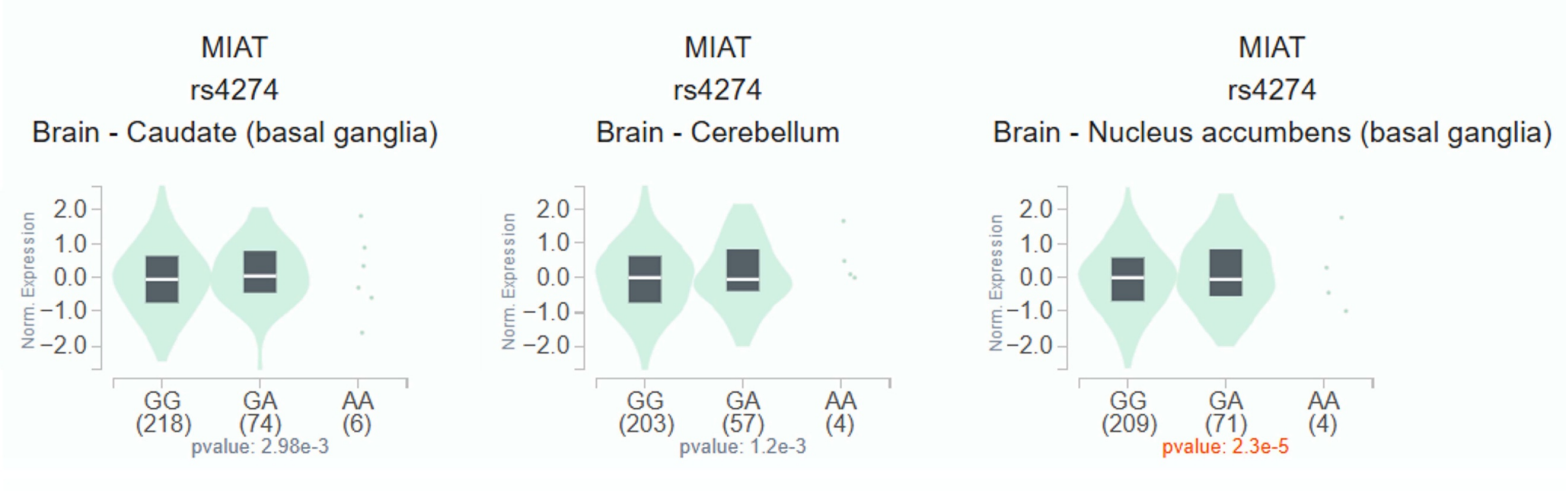
** Association of MIAT expression with *MIAT* rs4274 polymorphisms in GTEx dataset.** MIAT mRNA expression levels in brain tissue according to rs4274 genotypes, showing higher expression in carriers of the A allele.

**Figure 2:**
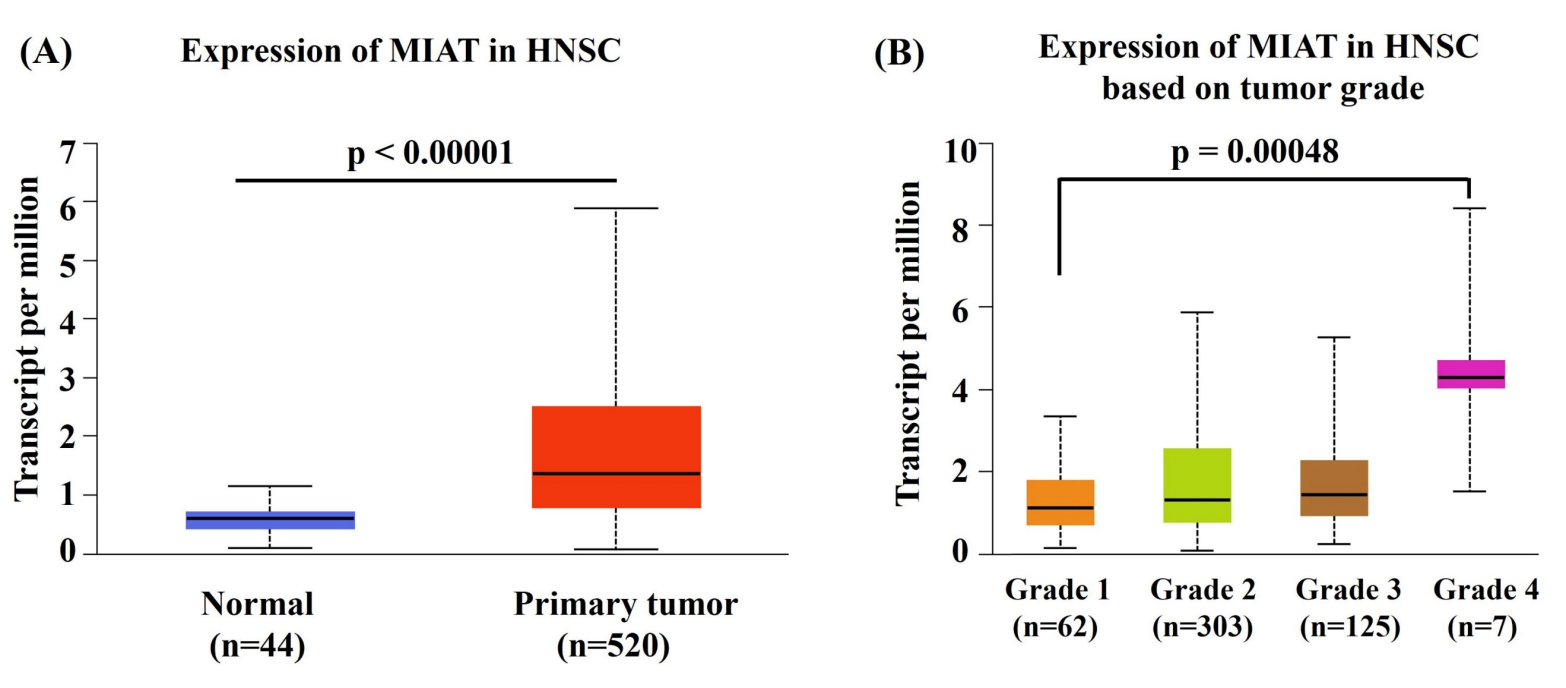
** MIAT expression patterns in head and neck squamous cell carcinoma.** (A) UALCAN analysis showing elevated MIAT expression in head and neck squamous cell carcinoma (HNSC) tissues compared with normal tissues. (B) Boxplot from the UALCAN database showing MIAT mRNA expression levels in HNSC tissues across different tumor grades.

To investigate the functional relevance of *MIAT* rs4274, we analyzed publicly available bioinformatic databases. GTEx analysis demonstrated that carriers of the rs4274 A allele exhibited significantly higher MIAT expression across various tissues compared to GG homozygotes (Figure 1). Analysis of TCGA data via the UALCAN platform revealed that MIAT expression was significantly elevated in HNSC tumor tissues relative to normal tissues (Figure 2A). Furthermore, higher MIAT mRNA levels were associated with increased tumor grade, with grade 4 tumors showing significantly greater expression compared to grade 1 tumors (p = 0.00048) (Figure 2B).

## Discussion

In this study, we investigated the associations between *MIAT* SNPs and male tongue cancer. Alcohol consumption, betel quid chewing, and tobacco smoking are well-established environmental risk factors contributing to tongue cancer initiation, development, and progression 29, 30. Oral tongue squamous cell carcinoma is considered the most common malignancy of the oral cavity 3, 31, 32. In our current study, statistically significant associations of these environmental risk factors including alcohol drinking, betel quid chewing, and cigarette smoking were found in 397 male patients with tongue cancer compared with 1194 controls, respectively. Although evidence linking alcohol consumption and betel quid chewing to MIAT expression is limited, a study on chronic obstructive pulmonary disease (COPD) reported that MIAT is upregulated in the lung tissues of cigarette smoke (CS)-exposed mice 33. Furthermore, MIAT knockdown attenuated CS- or CS-extract-induced inflammatory responses, collagen deposition, and EMT 33, suggesting a potential role of MIAT in mediating the effects of cigarette smoking and its contribution to disease progression.

We further examined the association for tongue cancer in relation to *MIAT* genotypic frequencies. Although previous studies have suggested a potential association between the lncRNA *MIAT* rs1061540 and ovarian cancer 14, and reported that *MIAT* rs1894720 variants are associated with various malignancies 16, 17, 19, 34. However, no significant associations were observed between these two polymorphisms and male tongue cancer in our study. This finding suggests a limited contribution of *MIAT* rs1061540 and rs1894720 polymorphisms to disease susceptibility and carcinogenesis in male tongue cancer. Additionally, previous research indicated that the rs4274 “AA” genotype may confer an increased risk of paranoid schizophrenia in the Chinese Han population 21. Consistent with this, our study found a statistically significant association between the *MIAT* rs4274 “AA” genotype and male tongue cancer, suggesting a potentially more prominent role for *MIAT* rs4274 polymorphisms in the pathogenesis and susceptibility of male tongue cancer.

We further analyzed the associations of *MIAT* rs4274 genotypic frequencies in 397 male tongue cancer patients. The *MIAT* rs4274 “GA+AA” genotype was significantly associated with moderate or poor tumor cell differentiation. Interestingly, when clinical status was analyzed in combination with environmental risk factors, a significant association was observed between moderate or poor differentiation and betel quid chewing. Betel quid chewing is recognized as a major carcinogenic risk factor for oral tongue cancer 30, 35, and MIAT has been suggested as an independent predictor of poor prognosis in OTSCC 10.

However, to date, no study has investigated the interaction between *MIAT* SNP and betel quid chewing in tongue cancer. In the present study, we observed that male tongue cancer patients who were betel quid chewers and carried the *MIAT* rs4274 “GA+AA” polymorphic variants had a significantly higher risk of moderate or poor tumor cell differentiation. Given that MIAT has been reported to promote oral cancer cell invasion by regulating EMT marker expression through Wnt/β-catenin pathway activation 10, 36, our findings suggest a potential synergistic effect of *MIAT* rs4274 “GA+AA” variants and betel quid chewing on tongue cancer susceptibility and progression. Nevertheless, the precise mechanisms underlying this interaction remain unclear.

Considering that the *MIAT* rs4274 “GA+AA” carriers exhibit moderate or poor cell differentiation, it is plausible that the “A” allele may upregulate MIAT expression independently of environmental risk factors. Previous studies have reported elevated MIAT levels in both oral cancer tissue and serum, implicating MIAT in tumor growth, migration, invasion, and EMT 10. Moreover, bioinformatic analyses also indicated that the *MIAT* rs4274 A allele is associated with increased MIAT expression, and higher MIAT levels correlated with higher tumor grade. Therefore, *MIAT* rs4274 “A” allele may contribute directly to oral tongue cancer progression.

For smokers carrying the *MIAT* rs4274 “A” allele, we observed a higher risk of moderate or poor differentiation. Supporting this, prior research has shown that MIAT mediates cigarette smoke-induced EMT and airway remodeling via the miR-29c-3p-HIF3A axis in COPD 33. Although these patients did not show a significantly higher risk of advanced clinical stage in our cohort, it is reasonable to propose that elevated MIAT expression may promote more aggressive tumor progression, potentially contributing to poor prognosis and increased cancer mortality. A limitation of our study is the absence of serum MIAT level data from the enrolled patients, which precludes a more comprehensive analysis. Future well-designed studies are warranted to elucidate the relationship between *MIAT* rs4274 variants and MIAT expression in oral tongue cancer, which may help identify MIAT as a potential biomarker for tongue cancer susceptibility and disease progression.

In conclusion, our study demonstrates associations between *MIAT* rs4274 polymorphisms and tongue cancer susceptibility, as well as clinical outcomes in relation to environmental risk factors. To our knowledge, this is the first study to report an interaction between betel quid chewing and the *MIAT* rs4274 “A” allele in promoting male tongue cancer progression. Specifically, betel quid chewers carrying the rs4274 “A” allele tended to develop moderate or poor cell differentiation, and smokers with the same genotype were at higher risk of poor differentiation. Collectively, *MIAT* rs4274 polymorphisms may serve as a pivotal biomarker for predicting MIAT-mediated tongue cancer susceptibility and progression in males.

## Figures and Tables

**Table 1:** The distributions of demographical characteristics in 1194 controls and 397 male patients with tongue cancer.

Variable	Controls (N=1194)	Patients (N=397)	p value
Age (yrs)			
< 55	564 (47.2%)	192 (48.4%)	p = 0.743
≥ 55	630 (52.8%)	205 (51.6%)	
Betel quid chewing			
No	996 (83.4%)	133 (33.5%)	
Yes	198 (16.6%)	264 (66.5%)	p < 0.001*
Cigarette smoking			
No	560 (46.9%)	90 (22.7%)	
Yes	634 (53.1%)	307 (77.3%)	p < 0.001*
Alcohol drinking			
No	957 (80.1%)	248 (62.5%)	
Yes	237 (19.9%)	149 (37.5%)	p < 0.001*
Stage			
I+II		179 (45.1%)	
III+IV		218 (54.9%)	
Tumor T status			
T1+T2		201 (50.6%)	
T3+T4		196 (49.4%)	
Lymph node status			
N0		244 (61.5%)	
N1+N2+N3		153 (38.5%)	
Metastasis			
M0		395 (99.5%)	
M1		2 (0.5%)	
Cell differentiation			
Well differentiated		48 (12.1%)	
Moderately or poorly differentiated		349 (87.9%)	

* p value < 0.05 as statistically significant.

**Table 2:** The adjusted odds ratio and 95% confidence interval (CI) of tongue cancer associated with *MIAT* genotypic frequencies.

Variable	Controls (N=1194) (%)	Patients (N=397) (%)	AOR (95% CI)	p value
**rs4274**				
GG	446 (37.3%)	162 (40.8%)	1.000 (reference)	
GA	555 (46.5%)	187 (47.1%)	0.997 (0.754-1.319)	p=0.985
AA	193 (16.2%)	48 (12.1%)	**0.609 (0.404-0.917)**	p=0.018*
GA+AA	748 (62.7%)	235 (59.2%)	0.887 (0.681-1.155)	p=0.373
**rs1061540**				
TT	397 (33.2%)	119 (30.0%)	1.000 (reference)	
TC	574 (48.1%)	200 (50.4%)	1.215 (0.904-1.633)	p=0.197
CC	223 (18.7%)	78 (19.6%)	1.179 (0.811-1.715)	p=0.388
TC+CC	797 (66.8%)	278 (70.0%)	1.205 (0.911-1.593)	p=0.191
**rs1894720**				
GG	514 (43.0%)	155 (39.0%)	1.000 (reference)	
GT	525 (44.0%)	191 (48.1%)	1.093 (0.828-1.422)	p=0.532
TT	155 (13.0%)	51 (12.9%)	0.984 (0.651-1.487)	p=0.937
GT+TT	680 (57.0%)	242 (61.0%)	1.068 (0.820-1.389)	p=0.626

The adjusted odds ratio (AOR) with their 95% confidence intervals were estimated by multiple logistic regression models after controlling for betel quid chewing, cigarette smoking, and alcohol drinking.

**Table 3:** Odds ratio (OR) and 95% confidence intervals (CI) of clinical statuses associated with genotypic frequencies of *MIAT* rs4274 in 397 male tongue cancer patients.

Variable	GG (N=162)	GA+AA (N=235)	OR (95% CI)	p value
**Clinical Stage**				
Stage I+II	78 (48.1%)	101 (43.0%)	1.000 (reference)	p=0.309
Stage III+IV	84 (51.9%)	134 (57.0%)	1.232 (0.824-1.842)	
**Tumor size**				
≤ T2	78 (48.1%)	123 (52.3%)	1.000 (reference)	p=0.418
> T2	84 (51.9%)	112 (47.7%)	0.846 (0.566-1.262)	
**Lymph node metastasis**				
No	104 (64.2%)	140 (59.6%)	1.000 (reference)	p=0.353
Yes	58 (35.8%)	95 (40.4%)	1.217 (0.805-1.840)	
**Metastasis**				
M0	161 (99.4%)	234 (99.6%)	1.000 (reference)	p=0.792
M1	1 (0.6%)	1 (0.4%)	0.688 (0.043-11.080)	
**Cell differentiated grade**				
Well	28 (17.3%)	20 (8.5%)	1.000 (reference)	p=0.001*
Moderate or poor	134 (82.7%)	215 (91.5%)	**2.246 (1.217-4.147)**	

The odds ratio (OR) with their 95% confidence intervals were estimated by logistic regression models. * p value < 0.05 as statistically significant.

**Table 4:** Clinical statuses and genotypic frequencies of *MIAT* rs4274 in 397 male tongue cancer patients who are betel quid chewers or not betel quid chewers.

	Non-Betel Quid Chewers (N=133)				Betel Quid Chewers (N=264)			
Variable	GG (N=47)	GA+AA (N=86)	OR (95% CI)	p value	GG (N=115)	GA+AA (N=149)	OR (95% CI)	p value
**Clinical Stage**								
Stage I+II	23 (48.9%)	36 (41.9%)	1.000 (reference)	0.433	55 (47.8%)	65 (43.6%)	1.000 (reference)	0.497
Stage III+IV	24 (51.1%)	50 (58.1%)	1.331 (0.651-2.720)		60 (52.2%)	84 (56.4%)	1.185 (0.727-1.931)	
**Tumor size**								
≦ T2	23 (48.9%)	46 (53.5%)	1.000 (reference)	0.616	55 (47.8%)	77 (51.7%)	1.000 (reference)	0.535
> T2	24 (51.1%)	40 (46.5%)	0.833 (0.409-1.698)		60 (52.2%)	72 (48.3%)	0.857 (0.527-1.395)	
**Lymph node metastasis**								
No	27 (57.4%)	49 (57.0%)	1.000 (reference)	0.958	77 (67.0%)	91 (61.1%)	1.000 (reference)	0.325
Yes	20 (42.6%)	37 (43.0%)	1.019 (0.497-2.092)		38 (33.0%)	58 (38.9%)	1.291 (0.776-2.149)	
**Cell differentiation**								
Well	6 (12.8%)	9 (10.5%)	1.000 (reference)	0.689	22 (19.1%)	11 (7.4%)	1.000 (reference)	0.006*
Moderate or poor	41 (87.2%)	77 (89.5%)	1.252 (0.417-3.762)		93 (80.9%)	138 (92.6%)	**2.968 (1.374-6.410)**	

The odds ratio (OR) with their 95% confidence intervals were estimated by logistic regression models. * p value < 0.05 as statistically significant.

**Table 5:** Clinical statuses and genotypic frequencies of* MIAT* rs4274 in 397 male tongue cancer patients who are smoker and non-smokers.

	Non-smoker (N=90)				Smoker (N=307)			
Variable	GG (N=29)	GA+AA (N=61)	OR (95% CI)	p value	GG (N=133)	GA+AA (N=174)	OR (95% CI)	p value
**Clinical Stage**								
Stage I+II	18 (62.1%)	23 (37.7%)	1.000 (reference)	0.033*	60 (45.1%)	78 (44.8%)	1.000 (reference)	0.960
Stage III+IV	11 (37.9%)	38 (62.3%)	**2.704 (1.087-6.726)**		73 (54.9%)	96 (55.2%)	1.012 (0.643-1.592)	
**Tumor size**								
≦ T2	14 (48.3%)	31 (50.8%)	1.000 (reference)	0.822	64 (48.1%)	92 (52.9%)	1.000 (reference)	0.409
> T2	15 (51.7%)	30 (49.2%)	0.903 (0.373-2.188)		69 (51.9%)	82 (47.1%)	0.827 (0.526-1.299)	
**Lymph node metastasis**								
No	20 (69.0%)	31 (50.8%)	1.000 (reference)	0.108	84 (63.2%)	109 (62.6%)	1.000 (reference)	0.926
Yes	9 (31.0%)	30 (49.2%)	2.151 (0.846-5.468)		49 (36.8%)	65 (37.4%)	1.022 (0.641-1.631)	
**Cell differentiation**								
Well	3 (10.3%)	7 (11.5%)	1.000 (reference)	0.873	25 (18.8%)	13 (7.5%)	1.000 (reference)	0.004*
Moderate or poor	26 (89.7%)	54 (88.5%)	0.890 (0.213-3.724)		108 (81.2%)	161 (92.5%)	**2.867 (1.405-5.849)**	

The odds ratio (OR) with their 95% confidence intervals were estimated by logistic regression models. * p value < 0.05 as statistically significant.
